# A primary care, multi-disciplinary disease management program for opioid-treated patients with chronic non-cancer pain and a high burden of psychiatric comorbidity

**DOI:** 10.1186/1472-6963-5-3

**Published:** 2005-01-13

**Authors:** Paul R Chelminski, Timothy J Ives, Katherine M Felix, Steven D Prakken, Thomas M Miller, J Stephen Perhac, Robert M Malone, Mary E Bryant, Darren A DeWalt, Michael P Pignone

**Affiliations:** 1 Department of Medicine, University of North Carolina at Chapel Hill School of Medicine, Chapel Hill, North Carolina, USA; 2 Division of Pharmacotherapy, University of North Carolina at Chapel Hill School of Pharmacy, Chapel Hill, North Carolina, USA; 3 Robert Wood Johnson Clinical Scholars Program, University of North Carolina at Chapel Hill School of Medicine, Chapel Hill, North Carolina, USA

## Abstract

**Background:**

Chronic non-cancer pain is a common problem that is often accompanied by psychiatric comorbidity and disability. The effectiveness of a multi-disciplinary pain management program was tested in a 3 month before and after trial.

**Methods:**

Providers in an academic general medicine clinic referred patients with chronic non-cancer pain for participation in a program that combined the skills of internists, clinical pharmacists, and a psychiatrist. Patients were either receiving opioids or being considered for opioid therapy. The intervention consisted of structured clinical assessments, monthly follow-up, pain contracts, medication titration, and psychiatric consultation. Pain, mood, and function were assessed at baseline and 3 months using the Brief Pain Inventory (BPI), the Center for Epidemiological Studies-Depression Scale scale (CESD) and the Pain Disability Index (PDI). Patients were monitored for substance misuse.

**Results:**

Eighty-five patients were enrolled. Mean age was 51 years, 60% were male, 78% were Caucasian, and 93% were receiving opioids. Baseline average pain was 6.5 on an 11 point scale. The average CESD score was 24.0, and the mean PDI score was 47.0. Sixty-three patients (73%) completed 3 month follow-up. Fifteen withdrew from the program after identification of substance misuse. Among those completing 3 month follow-up, the average pain score improved to 5.5 (p = 0.003). The mean PDI score improved to 39.3 (p < 0.001). Mean CESD score was reduced to 18.0 (p < 0.001), and the proportion of depressed patients fell from 79% to 54% (p = 0.003). Substance misuse was identified in 27 patients (32%).

**Conclusions:**

A primary care disease management program improved pain, depression, and disability scores over three months in a cohort of opioid-treated patients with chronic non-cancer pain. Substance misuse and depression were common, and many patients who had substance misuse identified left the program when they were no longer prescribed opioids. Effective care of patients with chronic pain should include rigorous assessment and treatment of these comorbid disorders and intensive efforts to insure follow up.

## Background

Chronic, non-cancer pain, defined as pain of greater than 3 months duration, is a common, important health issue. The prevalence of chronic pain ranges from 20% to 60% [1]. The prevalance of low back pain is greater than 30%[2], and the prevalance of migraine is approximately 15%[3,4]. Pain disorders, including headache, back pain, arthritis and other musculoskeletal pain, are estimated to cost the United States economy $61 billion per year in lost productive time [5]. It is frequently asserted that chronic non-cancer pain is undertreated [6-8].

The optimal approach to treating chronic pain is controversial [9-13]. The realization that acute pain and cancer pain were often undertreated led to a liberalization of opioid use in these populations in the late 1980's and throughout the 1990's [6,14-16]. Subsequently, many advocates and practitioners called for an increase in the use of opioids for patients with chronic non-cancer pain as well [14,17,18]. Other experts assert that use of opioids for chronic pain should be limited [10,19]). used with caution [20], or eschewed altogether due to potential for substance misuse and lack of proven efficacy [13].

In large part, uncertainty about how to best manage patients with chronic pain stems from a lack of research in this area [21]. The limited available research comes from studies performed in specialty practice settings [11,22-25]; few studies have examined the care of chronic pain in primary care [26,27]. Unlike other common chronic diseases such as congestive heart failure or diabetes, there are few clinical trials to inform practice guidelines.

Chronic pain management is complex. Like other chronic diseases, chronic pain is multi-faceted, and it is attended by its own set of comorbidities. It is complicated by substantial psychological and functional impairment in the forms of depression, disability, and loss of livelihood [28-30]. It is also costly. Patients with depression and back pain or migraine incur 3 to 4 times higher medical costs than patients with these pain conditions alone [31].

Generalists, even if well-versed in the biopsychosocial model of disease, often feel unprepared to manage chronic pain. They may lack the training in using certain pharmacological regimens, such as combining chronic opioids with psychiatric and pain modulating agents [27,32]. They may fear regulatory scrutiny and sanction when prescribing opioids, and they may be wary of fostering opioid dependence, misuse or addiction [33,34]. These barriers result in variations and inconsistencies in care that leave both patients and providers frustrated.

We developed a disease management program to improve the management of chronic pain in opioid-treated patients attending an academic general medicine practice. Traditional models of office-based care focus on diagnonsis and acute management of medical problems in the context of a single provider-patient relationship. In contrast, disease management emphasizes: (1.) The use of multi-disciplinary teams providing integrated care; (2.) Evidence-based algorithms; (3.) Interval visits to monitor response to therapy; and (4.) Information systems that permit tracking of outcomes and adjustment of therapy [35-39]. We applied these principles in developing and implementing an intensive, structured, and coordinated program to improve the management of patient with chronic pain. We focused our program on patients treated with opioid medications and attempted to provide an environment that would allow the safe and effective use of these drugs. We sought to determine if this program could improve pain, functional, and psychiatric outcomes in a 3 month uncontrolled trial.

## Methods

### Development of the program

In designing the program, we reviewed existing research, contacted experts, and conducted informal assessments of the main barriers to effective pain management in our practice [40]. Barriers existed at the provider and patient levels. These included part-time providers, frequent provider turnaround, physicians in training, and a socio-economically-disadvantaged, geographically dispersed patient population with multiple comorbidities. We applied lessons and systems from existing disease state management programs in diabetes, anticoagulation, and heart failure in our practice [41].

### Patient recruitment

Patients were eligible if they had pain of greater than 3 months duration and were either taking or considering opioid therapy. Attending and resident physicians were encouraged to refer patients if they were having difficulty managing their pain or if they suspected misuse of opioid medications. We publicized the program through educational conferences with residents and attendings and through informal communication within our practice.

### Baseline assessment

After obtaining informed consent, a research assistant administered a comprehensive baseline assessment to gather socio-demographic data and review the medical history with an emphasis on the medical management of pain. Validated measures of pain, disability and psychological status were included. Using an 11 point scale, the Brief Pain Inventory (BPI) asked patients to rate their pain at the time of the interview and at its worst, least, and average over the past month [42,43]. The 7 item Pain Disability Index (PDI), a measure of pain related disability, asked patients to rate the degree of disability on a 10 point scale [44-47]. Higher scores indicate higher disability and the scale discriminates between high and low levels of disability. To assess depression, we used the Center for Epidemiological Studies-Depression Scale (CESD) [48]. This twenty item tool rates affective symptoms on a scale of 0 to 3.

### Intervention

Patients were managed by a multi-disciplinary team consisting of the patient's primary care physician, a clinical pharmacist, a program assistant with training in health behavior, and a psychiatrist with sub-specialization in pain management who saw patients with the team one half-day per week. One nurse was dedicated to checking-in study patients and obtaining urine specimens. At entry, all patients signed a Medication Contract (Appendix A) [49] and provided a sample of urine for toxicological testing (UTS) [50,51]. The Medication Contract specified the conditions under which opioids would or would not be prescribed.

The clinical pharmacist or psychiatrist modified or titrated a patient's pain medications in consultation with the primary care physician. During titration of medications, patients returned at one month intervals. Medical management adhered to published guidelines and expert opinion on the management of chronic, non-cancer pain [17,52-54]. The principles of management were:

• Longer-acting opioids (long-acting morphine, fentanyl patches, methadone, sustained-release oxycodone) were initiated in patients who had been receiving short-acting agents that were only partially effective.

• Short-acting, potent opioids (usually oxycodone preparations) were prescribed for breakthrough pain.

• Longer-acting opioids were titrated at interval visits.

• Less costly, generic medications (e.g. methadone and long-acting morphine) were preferred over proprietary products of equal or lesser efficacy [55].

• Tricyclic anti-depressants, gabapentin, and other agents were used adjunctively, especially for neuropathic pain.

To address psychiatric comorbidity, patients with depression and other complex psychiatric conditions (e.g. psychotic depression and bipolar disorder with substance misuse) received psychiatric evaluation. Depression was diagnosed based on clinical interview and CESD score. In addition, primary care physicians could request psychiatric consultation on patients who had unaddressed psychiatric problems. The clinical pharmacist, psychiatrist and primary care physicians used CESD scores to guide treatment of patients scoring in the depressed range. The program, however, did not employ a strict protocol for depression treatment.

As defined in our medication agreement with participants, we prospectively monitored substance misuse through clinical history, review of medications, communication with pharmacies and providers, and urine toxicological screening (UTS). Medications were documented in the electronic medical record and our program database. Discrepencies and inconsistencies were discussed with the patient's primary provider. We contacted a patient's pharmacy to verify procurement of medications, and, if substance misuse was suspected, we contacted additional pharmacies to ascertain whether or not a patient was receiving opioids from multiple sources. A UTS was obtained at each visit and was correlated with the patient's reported history of medication use. In collaboration with our institution's toxicologist, results of the UTS were verified using the appropriate confirmatory assays. For example, the presence or absence of "opiates" on the UTS was confirmed using gas chromatography. In addition, all positive results for amphetamines were confirmed with gas chromatography due to the possibility of assay interference from other medications [50,51].

We defined serious substance misuse prospectively as any of the following: 1. Cocaine or amphetamines detected on UTS; 2. Procurement of opioids from more than one provider on a regular basis ("doctor collecting"); 3. Diversion of opioids; 4. UTS negative on at least two occasions for prescribed opioids in the context of a reported history that the patient was taking the medication as prescribed (We considered repeatedly "negative" urines as an indicator of possible diversion.); 5. UTS positive on at least two occasions for opioids not prescribed by our practice (an inappropriate or inconsistent urine). A positive cannabinoid finding on UTS was not defined as serious substance misuse for the purposes of our study, but we counseled patients to refrain from marijuana use.

Patients were advised at entry into our program (and in the Medication Contract) that serious violations of the contract would result in discontinuation of opioids. Past instances of serious misuse were not subject to sanction. We constituted a formal practice-wide committee to evaluate and respond to suspected misuse. It consisted of the practice director, two attending physicians, a clinical pharmacist, two resident physicians, and a nurse. The committee deliberated through secure email and considered the violations defined above. Patients committing serious substance misuse were offered referral to substance abuse experts at our institution. In most cases, opioid therapy would be reconsidered in 6 months if the patient participated in substance abuse counseling (The practice policy addressing substance misuse is included as Appendix B.).

### Reassessment

At 3 month follow-up, a research assistant reassessed each patient's clinical status. Pain, disability, and mood scores were re-measured using the instruments previously described. The research assistant was not blinded to study participation status.

### Analysis

Descriptive statistics are reported as means and percents. Paired t-tests were used to compare changes in pain, disability and depression scores from baseline to 3 month follow-up. McNemar's test was used to measure differences in proportions of patients receiving treatment for depression at 0 and 3 months. We also compared changes in pain scores based on changes in opioid dose. All analyses were performed using Stata 7.0 (College Station, TX).

The research protocol was approved by the University of North Carolina School of Medicine Committee on the Protection of the Rights of Human Subjects. The funding sources had no role in the collection or interpretation of the results.

## Results

Between December 2002 and May 2003, 85 patients agreed to participate in the study. Table 1 presents the baseline demographic characteristics of the study participants. All patients completed baseline assessment and 63 (73%) completed the 3 month assessment. Of the 22 patients who did not complete 3 month assessment, 15 did not return after a serious violation of the medication contract led to the discontinuation of opioids, 4 patients were lost to follow up, and 3 changed their venue of primary care. There were no important differences in baseline demographic, pain, depression, or disability scores between completers (n = 63) and all non-completers (n == 22), although some differences emerged among non-completers who committed substance misuse (n = 15) (Table 2.).

**Table 1:** Univariate Analysis

	**N = 85**
Mean age, y (± SD)	51 (9.6)
Range	27–76
Male, %	60
White Race, %	78
Marital Status, %	
Married	49
Stable relationship	7
Unmarried	44
Disabled, %	65
Education, %	
Not high school graduate	38
HS graduate	28
Some college	34
Income <$20,000/yr, %	83
Medicare or Medicaid, %	58
Uninsured,%	29
History of Smoking, %	87
H/O Alcohol Use, %	75
H/O Substance Use, %	44
H/O Depression, %	51

**Table 2:** Characteristics of Study Completers and Non-Completers

**Characteristic**	**Completers (N = 63)**	**Non-Completers (N = 22)**	**P-Value**	**Non-Completers with Substance Misuse (N = 15)**	**P-Value**
Age, y	51	49	0.422	48	0.215
% Male	62	55	0.544	67	0.732
% White	81	68	0.216	60	0.083
% High School Graduate	62	62	0.975	64	0.890
% Disabled	66	63	0.815	54	0.405
% Uninsured	29	32	0.774	33	0.716
% Substance Use	40	55	0.226	67	0.059
CESD	24	27	0.416	31	0.050
PDI	47	41	0.141	46	0.810
Pain Scores					
Worst in last month	9.2	9.3	0.727	9.2	0.946
Least in last month	4.6	4.4	0.768	4.7	0.884
Average in last month	6.5	6.5	0.925	6.6	0.861
Current pain	6.8	7.3	0.361	7.5	0.279

### Patient characteristics

The average age of patients was 51 years, 60% were male, and 78% were white, most (83%) had an income less than $20,000 per year and 65% were disabled. Forty-four percent had a history of illicit substance use (e.g. marijuana, cocaine, amphetamines). All patients had pain of at least 3 months duration and 90% had pain for greater than 1 year. At baseline 93% were receiving opioids. At 3 month follow up, 97% were receiving opioids.

Table 3 presents the principal pain types. The lumbar spine was the most frequently involved primary site. Overall, axial spine pain accounted for 49% of the primary pain reported by patients. Myofascial pain and polyarticular arthritis were also frequently represented. Patients in the "Other" category commonly had mixed etiologies of pain, often attributable to previous trauma or surgery. One chronic headache patient is included in this category.

**Table 3:** Primary Pain Type (N = 85)

	**Number (%)**
Spine	42 (49)
Lumbar	30 (35)
Cervical	7 (8)
Thoracic	5 (6)
Diffuse (Fibromyalgia, Chronic fatigue syndrome)	13 (15)
Polyarthritis	8 (9)
Knee	5 (6)
Abdomen	4 (5)
Diffuse neuropathic	4 (5)
Elbow & Hip	2 (2)
Other	7 (8)

### Effect of the intervention

Table 4 presents the effect of the intervention on pain, functional status and depression. Baseline results reveal high pain scores. The worst pain was 9.2, the least pain was 4.6, the average pain was 6.5, and current pain was 6.8. The average PDI score, 47.0, suggested substantial disability. There was a high prevalence of depression. The mean CESD score, 24.0, falls in the "severely depressed" category of the scale.

**Table 4:** Pre and Post Intervention (N = 63)

	**Pre**	**Post**	**Improvement, %**	P-Value*
Pain at worst in the last month^&^	9.2	8.1	12	<0.001
Pain at least during the last month	4.6	3.9	15	0.038
Pain on average during the last month	6.5	5.5	15	0.003
Pain right now	6.8	5.8	15	0.014
Pain Disability Index	47.0	39.3	16	<0.001
CESD	24.0	18.0	25	<0.001
% CESD in depression range:				
Conventional cutoffs^£^	79.4	54.0	32	0.003
Chronic pain cuttoffs^¶^	38.1	23.8	37	0.049
% Depression medication	44.4	52.4	15	0.059

* Paired t-test except where indicated

McNemar's test

^&^Score 1–3 is mild pain; 4–6, moderate pain; 7–10, severe pain

£ Score of ≥ 15

^¶^Score of ≥ 27

At 3 month follow up, BPI pain scores improved by 12% to 15%, and all reductions were statistically significant. The mean depression score improved from 24.0 to 18.0 (p < 0.001), and the proportion of patients scoring in the depression range decreased from 79% to 54% (p = 0.003). We did not correct for multiple comparisons because of the exploratory nature of our analyses.

Some authors have demonstrated that the conventional cutoffs for the CESD (depression ≥ 15; severe depression ≥ 22) may lack specificity in patients with chronic pain and have proposed a CESD threshold of 27 for diagnosing depression in this population [56]. Using this threshold, the proportion of patients scoring in the depressed range decreased from 38% to 24% (p = 0.049). Pharmacologically, depression was undertreated at baseline. The proportion of depressed patients receiving anti-depressants increased from 44% at baseline to 52% at 3 months (p= 0.059).

### Relationship between pain and opioid dosing

The mean daily opioid dose in milligram equivalents of morphine increased from 72 mg per day to 91 mg per day (A milligram morphine equivalent approximates a milligram of oxycodone.). Forty-eight percent of patients had their opioid dose increased over 3 months. In these patients, the mean opioid equivalent increased from 53 mg to 105 mg per day. No clear relationship emerged between opioid dosing and improvements in pain, disability, and depression scores, after adjusting for baseline pain, disability and depression (Table 5.).

**Table 5:** Effect of Opioid Increase on Pain (N = 63)

	**Opioids Increased**		**P-Value**
		
	**Yes (n = 30)**	**No (n = 33)**	
Δ Pain at worst in the last month	1.40	0.99	0.37*
Δ Pain at least during the last month	0.80	0.52	0.66*
Δ Pain on average during the last month	0.96	0.94	0.93*
Δ Pain right now	1.14	0.87	0.70*
Δ Pain Disability Index	8.34	7.03	0.63^¶^
Δ CESD	5.21	6.71	0.74

* Adjusted for baseline pain

¶Adjusted for baseline PDI

Adjusted for baseline CESD

### Substance misuse

Twenty-seven patients (32%) committed some form of serious substance misuse (Table 6.). Although we confirmed only one instance of diversion, we suspect that patients with repeatedly negative UTS's or inconsistent UTS's may have been diverting their medications. Substance misusers accounted for the preponderance of subjects who dropped out of the study. Table 2 compares selected baseline characteristics between substance misusers who did not complete three months and subjects who completed the trial. Although the numbers are small, there is a trend toward greater representation of non-white race, history of illicit substance use, worse depression, and higher pain scores at baseline assessment among substance misusers who did not complete the trial.

**Table 6:** Substance Misuse (N = 27)

**Misuse**	**Number (%)**
Stimulants on UTS	13 (15)
Cocaine	11 (14)
Amphetamines	2 (2)
Diversion	1 (1)
Doctor collecting	3(3)
Inappropriate/Inconsistent UTS	2 (2)
Negative ("Clean") Urines	7 (8)
Prescription adulteration	1 (1)

## Discussion

We found that a multi-disciplinary, primary care-based, disease management program can improve pain, depression and disability scores in opioid-treated patients with chronic pain in a 3 month uncontrolled trial. The improvements across all outcomes support an improved quality of life resulting from the intervention. These improvements were obtained using an approach that balanced the potential benefits and adverse effects of opioids.

We hyothesize that these improvements resulted from the combined effects of systematizing pain management and treating depression. Improvements appear independent of opioid dosing. The clinical significance of the 12% to 15% improvement in pain scores is unclear. Uncontrolled trials in specialty pain clinics have reported a 20% to 25% reduction in pain scores [57]. Some research suggests that a 30% decrease in pain scores (i.e. about 2 points) represents clinically significant relief of pain [58], but the issue of how to interpret pain scales clinically is not resolved.

The improvement in depression scores was clinically important and may reflect combined effects of intensification of pharmacological management for depression and pain and the systematization of care. Although the reciprocal relationship between pain and depression has been established in previous studies, the effect that treating one condition has on the other has not been well-assessed [29]. One recent study demonstrated that the presence of severe pain predicted a poor response to antidepressant therapy, and thus it is plausible that intensifying pain management would have a beneficial effect on depression [59]. Clearly, the improvements in depression scores seen in this study cannot be attributed to increasing anti-depression pharmacological therapy alone because the proportion of patients treated with anti-depressants increased from 44% to 52% only. We have since added a structured treatment algorithm to increase the use of anti-depressants. It is important to note that there was a statistically signficant trend toward greater depression among substance misusers who did not complete the trial; thus, it is possible that the trial overestimates the effect of multi-disciplinary management on depression outcomes.

To our knowledge, this is the first study to prospectively examine the effects of multi-discplinary pain management on the outcomes of pain, disability, depression, and substance misuse in an academic primary care practice caring for a wide range of patients. Previous studies conducted in a military clinic[60] and a health maintenance organization [26] demonstrated improved pain and functional scores with systematic, multidisciplinary intervention. The military trial was uncontrolled and enrolled referred patients into a specialty clinic. The HMO trial was conducted in a primary care setting. It was controlled, and did evaluate pain, function and mood outcomes. Neither study systematically monitored substance misuse.

We documented a high prevalence of substance misuse (32%). We did not assess addiction per se. The prevalence of substance misuse and addiction in patients receiving chronic opioids is unclear and depends on the populaton under study. Some authors have asserted that addiction and substance misuse are uncommon consequences of opioid use for pain. One widely cited reference estimated the prevalence of addiction at approximately 4 in 10,000 treated patients [61]. Others have reported prevalences of addiction ranging from 3% to 17% [62,63]. A recent retrospective study in a primary care setting documented a high prevalence of opioid misuse: 24% in a resident physician clinic and 31% in a Veterans Administration outpatient clinic[64], but the criteria used to determine the prevalence of opioid misuse were limited by chart review. Some behaviors defined as indicators of opioid misuse (e.g. lost or stolen medications, requests for early refills) could be construed as indicators of inadequately treated pain (i.e. "pseudoaddiction"[65]) and not substance misuse or addiction.

Opioid misuse not only complicates the management of pain in the individual patient, but has negative societal consequences as well, especially when opioids are diverted from their intended use [66-68]. Several states have documented increases in unintentional deaths from opioids, especially diverted methadone [69-71]. National surveys demonstrate dramatic increases in the non-medical use of OxyContin^® ^and other prescription drugs among teens and young adults [72,73]. The trauma literature has documented recent increases in opioid use among patients admitted to trauma centers [74]. In response, there have been state and national initiatives to reduce prescription drug misuse [75,76]. Though high rates of substance misuse are a source of concern, our program may serve as an example for how care can be organized to reduce misuse without eschewing the benefits of opioid medications.

Although the prevalence of substance misuse in our study population is higher than reported in clinical trials of opioids, these trials have occurred in specialty settings with selected populations. They excluded patients with a history of substance misuse, or have not systematically monitored patients for substance misuse [11,12,23,66,77]. In addition, they commonly excluded patients with psychiatric illness (including depression) which is a strong predictor of substance misuse [23,66,78-80]. Patients in our program had a high baseline prevalence of depression, previous substance and alcohol abuse, and other psychiatric disorders (Table 1.). The strong relationship between mental illness and substance abuse disorders is well known and thus the high prevalence of substance misuse is not entirely unexpected [81]. Previous studies of mental illness have documented a high coexisting prevalence of substance and alcohol misuse: 32% with unipolar depression; 61% with bipolar depression; 47% with schizophrenia; 84% with personality disorders: and 24% with anxiety disorders [78,82].

We specifically sought out patients whose pain management was difficult for providers or in whom substance misuse was suspected. Many had established or suspected psychiatric diagnoses. How to identify chronic pain patients at risk for drug misuse and to treat their pain remains a challenge [83-89]. The pattern of substance misuse in our population often suggested polysubstance abuse; this places patients at especially high risk of morbidity and mortality [90]. Although patients committing substance misuse were offered substance abuse treatment referral, only two followed through and most did not return to our program. The option of pain management without the use of opioid analgesics was offered to all patients who committed substance misuse.

The difficulty in obtaining mental health and substance abuse treatment services is a pressing public health issue and a topic of national debate in the United States [81]. In our sample there was a clear trend toward increased comorbid depression among substance misusers who did not complete three month follow up (Table 2.). Despite the availability of on-site psychiatric consultation, our program was not successful in retaining and managing a challenging subset of patients with substance misuse and depression.

We are aware that some of our substance misusing patients migrated to other practices in order to obtain opioids and other controlled substances. Our program implemented policies to prevent migration of patients *within *our practice (Appendix B.) and the University of North Carolina Health Care System. The cornerstone of these policies was meticulous documentation in an electronic medical record that is accessible to all physicians at our medical center and to hospitals and physicians affiliated with our system in the surrounding communities. In general, though, we have no direct control over migration that occurs *outside *of our practice and our health care system. In order to curtail migration and "doctor shopping," some states have implemented centralized monitoring systems for opioids and other controlled substances. North Carolina is currently exploring the feasibility of such a system. A description of the operational state monitoring programs is available online through the United States Drug Enforcement Agency Diversion Control Program website [91].

We believe that our results may be generalizable to other academic primary care practices that serve diverse patient populations with a high burden of medical and psychiatric comorbidity. The etiologies and sites of pain were similar to those reported in population-based epidemiological surveys and clinical trials in primary care, except that headache was under-represented in our population [1,12,92]. The results replicate epidemiological research that demonstrates a strong interaction between pain and the psychiatric comorbidities of depression and substance misuse [87,88].

Our study may be more applicable to the general medical setting than previous trials examining the effects of opioids on chronic pain because we did not exclude patients with serious psychiatric comorbidity or those suspected of substance abuse. To be effective, pain management should encompass more than pharmacological management directed at pain scores; it should address a variety of behavioral and psychosocial factors that contribute to suffering [86,93].

Our study has several limitations. It was uncontrolled and of relatively short duration. The research assistants were not blinded to pre- and post-treatment assessments. The improvements could reflect secular trends, although the chronic nature of our patients' symptoms and disability makes this less likely, and improvements were noted across all of the pre-specified outcomes. Not all patients receiving chronic opioids in our practice were referred. Thus, we may have over-estimated the prevalence of substance misuse because providers were more likely to refer "problem" patients in whom they suspected opioid misuse.

Another limitation of our study is its individualized nature. We did not adhere to strict algorithms for diagnosis and treatment and did not test a single intervention. The evidence-base for managing chronic pain in the general medicine setting is limited and the multi-modal nature of our intervention was by necessity empirical and exploratory. As such, we decided to allow more latitude and individualization in treatment choice. We have used our experience and the data collected to develop more robust algorithms to guide the management of pain and depression and to make psychiatric referral when appropriate.

As a corollary to our multi-modal approach, it is difficult to ascertain if the improvements derived from pain medications, intensification of depression therapy, or simply participation in an organized program. Improvements and changes in behavior that occur as a result of becoming a target of special interest in a program are often referred to as a Hawthorne effect [94,95].

## Conclusions

In a 3 month trial conducted in an academic primary care setting, a systematic, multi-disciplinary approach to chronic pain management that included the use of opioids and tools to prevent misuse was effective in improving pain, depression, and function scores. Future efforts will be directed at examining their durability and promoting their sustainability. A randomized control trial would determine whether these are real effects or represent a secular trend.

Comorbid depression and substance misuse were common. Efforts will also be made to further characterize the interaction of these and other comorbid psychiatric conditions with chronic pain. Chronic pain patients with substance abuse are a challenging subset of patients who could benefit from new research and initiatives to mitigate the risk of abuse while ameliorating pain control.

## Competing interests

The author(s) declare that they have no competing interests.

## Authors' contributions

PC developed the study designand intervention, performed the statistical analyses, and drafted the manuscript.TI developed the study designand intervention, administered surveys and directed pharmacologic management, assisted in editing and revising themanuscript.KF participated in developing the study design, administering surveys, data and projectmanagement, and editing and revising the manuscript. SP participated in study design, developing pharmacologic protocols, performing psychiatric evaluations, and editing and revising the manuscript.TM participated in developing the study design and editing and revising the manuscript.JP assisted in administering surveys, data management and collection, and editing and revising the manuscript.RM participated in the development of the study design, data management, editing and revising the manuscript.MB participated in the development of the study design, editing and revising the manuscript. DD provided statistical analytical support and assisted in the drafting, editing, and revising of the document.MP developed the study design, supervised the overall conduction of the study, participated in data analysis, and assisted in the drafting, editing and revising of the manuscript.

## Appendix A: Medication Contract

Patient Name ____________________________ Diagnosis ______________________________

Physician Name __________________________ Telephone Number _______________________

I agree to abide by the following guidelines for managing my prescription for opiate pain medications:

1. I will only request and receive opiate (narcotic) pain medications from Dr. _________ or from his/her designee in the Internal Medicine Clinic Pain Service. I agree to inform any other physicians participating in my care of this agreement. If another physician wishes to suggest changes in pain management, they can contact Dr. ___________ during regular business hours, but no changes will be made without such contact.

2. Dr. ____________ and I have agreed that I will receive the following:

medicine ______________, directions __________ quantity _____, per ___ days,

medicine ______________, directions __________ quantity _____, per ___ days,

medicine ______________, directions __________ quantity _____, per ___ days.

I will not request refills prior to this date. I understand that if my medicines are lost or stolen, they will not be refilled prior to the next refill date. If I use up my supply of medication before the date of the next refill, I understand that my doctor will not provide extra medication. I further understand that I may suffer symptoms of withdrawal. I will inform my doctor in a timely manner if I miss taking a dose of my medication, have an increased need for the pain medication, or have difficulty taking the medication as prescribed. If I find that the current dose of pain medication is no longer adequate, I will discuss this situation with my doctor at a scheduled visit.

3. I agree to use _________________________________________________Pharmacy, located at _____________________________________________________________, telephone ________________________, for filling prescriptions for all of my pain medicine.

4. I will bring all unused pain medicine to every office visit, including all current prescription vials.

5. While this contract is in effect I will not abuse alcohol or other illicit drugs. As a part of this program, urine drug screening will occur at enrollment and may be required at future visits.

6. I will not sell or share opiate medications.

7. If I violate the terms of this contract, I understand that my doctor and other doctors in the Internal Medicine Clinic will no longer prescribe opiate medications for me. If this occurs, I understand that I may receive care elsewhere or continue with my current doctor and not receive opiate medicines. If I change doctors, I agree to allow my current physician to contact my new physician to transfer medical information including information about chronic pain treatment.

8. I understand that my doctor may verify whether or not I have a history of criminal drug convictions.

Patient Signature ________________________________________________

(print name) ________________________________________________

Physician signature ________________________________________________

Date ________________________________________________

## Appendix B

UNC General Internal Medicine Practice

Pain Review Committee:

Policy on Serious Opioid or Controlled Substance Misuse & Misconduct

Definition

The Pain Review Committee defines serious misuse or misconduct with regard to opioid medications and other controlled substances as any of the following:

1. Forgery or alteration of prescriptions.

2. Use of cocaine or other stimulant medications (e.g. amphetamines) concurrently with prescribed opioids and their detection on urine toxicological testing. Stimulant abuse is strongly indicative of polysubstance abuse.

3. Diversion of opioids or controlled substances.

4. Doctor collecting or shopping: Procuring controlled substances from more than one provider and misrepresenting the fact. This is a felony in North Carolina.

5. Negative or "clean" urines: The absence of prescribed opioids from urine toxicological testing on at least two occasions in the context of a history that the patient is taking the medication as directed. This finding suggests diversion and/or substituting a separate urine sample.

6. Inappropriate or inconsistent urines: The presence on urine toxicological testing of opioids or other controlled substances (excluding cannabinoids) not prescribed by our clinic or pain program on at least two occasions without a reasonable explanation. This finding suggests polysubstance abuse, doctor collecting, or drug bartering (a form of diversion).

Procedure

Serious misuse or misconduct is a special category of misuse. It results in the immediate discontinuation of opioids in the Internal Medicine Clinic. The Pain Review Committee will address instances of serious misuse on an expedited basis. Individual cases will not require the review of the entire committee. The following procedure applies:

• The specific violation will be documented in the electronic medical record.

• Instances of serious misuse discovered by the pain management team will be discussed with the patient's primary care provider (PCP).

• The provider who discovers the violation will report it to the Clinic Director, Dr. Thomas Miller, or designee on the Pain Review Committee. (The designee will be either Dr. Paul Chelminski or Dr. Timothy Ives.) The designee will inform Dr. Miller of the violation and make a recommendation to Dr. Thomas Miller and the entire Pain Review Committee for the immediate discontinuation of opioids.

• Dr. Miller will make the final disposition on the recommendation.

• Committee members will receive communication about recommendations and disposition through email. Committee members may recommend alternative sanctions.

• The PCP will be informed of the disposition.

• The patient will receive verbal and written notice of disposition.

Sanctions

A. Serious misuse or misconduct will lead to one of two possible sanctions:

1. Forgery or alteration of prescriptions and diversion will result in immediate and permanent discontinuation of opioids and/or other controlled substances. The clinic director will decide whether instances of forgery or diversion also merit dismissal from the clinic.

2. Stimulant use, doctor collecting/shopping, negative urines, or inappropriate urines will result in immediate discontinuation of opioids and/or other controlled substances with possible re-evaluation in six months for a first violation. The Committee will stipulate substance abuse counseling as a condition for re-evaluation.

B. Two serious violations of the medication contract will result in permanent discontinuation of controlled substances.

Provider Issues

1. The Pain Review Committee cannot compel attending physicians to cease prescribing opioids or other controlled substances; however, providers who continue to prescribe these medications must understand that this practice may jeopardize their DEA license and/or expose them to regulatory and even criminal investigation. If the attending continues to prescribe opioids after a recommendation of discontinuation by the Committee, the patient is not eligible for re-enrolment in the General Internal Medicine Pain Program after six months.

2. The responsibility for stewardship and teaching of resident physicians requires special oversight of residents' patients who receive controlled substances. The residency program has an obligation to promote appropriate clinical practice and protect residents from practices that may jeopardize their professional status. In addition, residents prescribe scheduled substances under the authority of the hospital's DEA number, and the inappropriate prescription of opioids may expose the hospital to sanction. If the committee recommends discontinuation of opioids for the patient of a resident, the committee will instruct the resident that he or she can no longer prescribe scheduled medications for this patient. Likewise, other residents in the practice are not authorized to prescribe opioids to patients of residents or attendings who have had opioids discontinued.

Communication of Decisions

1. The patient should be informed of discontinuation verbally. Usually, this responsibility will fall to the PCP, but in certain instances it may fall to the person discovering the violation (e.g. the pharmacist who sees the patient in clinic for follow up in the pain program).

2. Dr. Miller will send the patient a registered letter.

3. The PCP will be copied on the letter.

4. A copy of the letter will be entered into the permanent electronic medical record as a Phone Message.

5. The patient's problem list on the electronic medical record will contain the entry "VIOLATION OF MEDICATION CONTRACT" and will be annotated "PATIENT VIOLATED THE MEDICATION CONTRACT SIGNED WITH GENERAL MEDICINE, AND WAS DISMISSED FROM NARCOTICS – SEE [date] CIS NOTE."

## Pre-publication history

The pre-publication history for this paper can be accessed here:


